# Combining social cognitive treatment, cognitive remediation, and functional skills training in schizophrenia: a randomized controlled trial

**DOI:** 10.1038/npjschz.2016.37

**Published:** 2016-11-09

**Authors:** Javier Peña, Naroa Ibarretxe-Bilbao, Pedro Sánchez, Maria B Iriarte, Edorta Elizagarate, Maria A Garay, Miguel Gutiérrez, Aránzazu Iribarren, Natalia Ojeda

**Affiliations:** 1 Department of Methods and Experimental Psychology, Faculty of Psychology and Education, University of Deusto, Bilbao, Spain; 2 Refractory Psychosis Unit, Hospital Psiquiátrico de Alava, C/Alava, Vitoria, Spain; 3 Department of Neuroscience, Psychiatry Section, School of Medicine and Odontology, University of the Basque Country, Vizcaya, Spain; 4 Red de Salud Mental de Bizkaia, Bilbao, Osakidetza; 5 CIBERSAM, Centro de Investigación Biomédica en Red de Salud Mental, Madrid, Spain; 6 Department of Psychiatry, Hospital Universitario Alava-Sede Santiago, C/Olaguibel, Vitoria, Spain

## Abstract

This study examined the efficacy of an integrative cognitive remediation program (REHACOP) in improving cognition and functional outcome in patients with schizophrenia. The program combines cognitive remediation, social cognitive intervention, and functional skills training. Few studies have attempted this approach. One hundred and eleven patients diagnosed with schizophrenia were randomly assigned to either the cognitive remediation group (REHACOP) or an active control group (occupational activities) for 4 months (three sessions per week, 90 min). Primary outcomes were change on general neurocognitive performance and social cognition, including theory of mind (ToM), emotion perception (EP), attributional style, and social perception (SP). Secondary outcomes included changes on clinical symptoms (Positive and Negative Syndrome Scale) and functional outcome (UCSD Performance-Based Skills Assessment and the Global Assessment of Functioning). The trial was registered with clinicaltrials.gov (NCT02796417). No baseline group differences were found. Significant differences were found in the mean change between the REHACOP group and control group in neurocognition ($\eta_{p}^{2}=0.138$), SP ($\eta_{p}^{2}=0.082$), ToM ($\eta_{p}^{2}=0.148$), EP ($\eta_{p}^{2}=0.071$), negative symptoms ($\eta_{p}^{2}=0.082$), emotional distress ($\eta_{p}^{2}=0.136$), Global Assessment of Functioning ($\eta_{p}^{2}=0.081$), and UCSD Performance-Based Skills Assessment ($\eta_{p}^{2}=0.154$). The combination of cognitive remediation, social cognitive intervention, and functional skills training demonstrated statistically significant and clinically meaningful changes in neurocognition, social cognition, negative, and functional disability.

## Introduction

Cognitive impairment has been considered a core feature of schizophrenia and it has been found in the prodomal phase of the disease^1^ and in unaffected relatives of patients with schizophrenia.^2^ Although not included in the diagnostic criteria for schizophrenia, the impairment of both basic cognition and social cognition is a well-established feature of schizophrenia.^3^

As suggested by several authors,^4,5^ social cognition comprises four main subdomains, including emotion perception (EP), theory of mind (ToM), social perception (SP), and attributional style (AS). Along with cognitive deficits,^2^ social cognitive deficits^6^ have been suggested as putative endophenotypes for schizophrenia. In addition, there is a large amount of evidence suggesting that cognitive deficits^7^ and social cognitive deficits,^4^ along with negative symptoms^8^ are linked to functional outcome in schizophrenia. Altogether, these results emphasize the importance of addressing cognition, social cognition, and negative symptoms in our treatments if our ultimate goal includes improving the functional outcome and quality of life of patients with schizophrenia.^9^

Considering the amount of evidence that has shown the link between neurocognition, and especially social cognition with regard to functional outcome, several groups have developed new treatments to improve basic cognitive deficits, as well as social cognition (see recent meta-analyses for review^10,11^). In this context, there is evidence of improvement after cognitive remediation by itself.^11^ Similarly, the same authors^10^ recently conducted a meta-analysis of social cognitive remediation studies. They found that social cognitive remediation induced improvements in ToM (*d*=0.70), facial affect recognition skills (*d*=0.84), aggression, blame, and hostility from AS (*d*=0.30, 0.48, and 0.52, respectively), SP (*d*=1.29), negative symptoms (*d*=0.32), and general symptoms (0.40).

More recently, some authors combined cognitive remediation with social cognitive remediation^9,12,13^ obtaining better results than patients receiving cognitive remediation alone in several domains. There is additional evidence that the combination of different trainings reciprocally boosts the effects of the treatments. For example, cognitive remediation combined with functional skill training improves functional outcome.^12,14–16^ More specifically, some authors found significant improvements in community activities and work skills,^14^ personal and social functioning,^17^ vocational outcome,^16,18^ and social adjustment.^19^

Given the evidence of the benefits of combining different training approaches, and especially the link between social cognition and functional outcome, we hypothesize that this integrative program (that combines cognitive remediation, social cognitive intervention, and functional skills training) will show significant improvements in several domains.

Therefore, the primary outcomes were change on general neurocognitive performance and social cognition. Secondary outcomes included changes on clinical symptoms and functional outcome.

## Results

**Figure 1:**
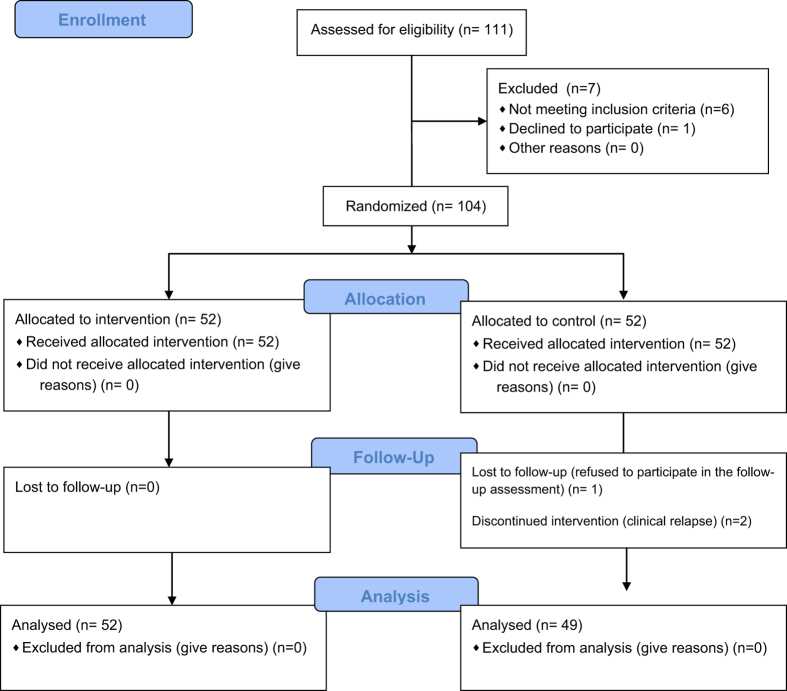
CONSORT flow diagram.

One hundred and one patients completed the post-test assessment, which reflects an attrition rate of 2.89% (Figure 1). The sociodemographic and clinical characteristics of the REHACOP and control group are provided in Table 1. There were no significant differences between the groups in terms of age, gender distribution, years of education, cognitive reserve, age at the onset, and/or number of hospitalizations (Table 1).

The attendance ranged between 96 and 100%. When a patient missed a session or several for different reasons (i.e., relevant change in the clinical treatment, vacations, or permission) he/she received individual training based on the contents trained by the group. Therefore, the patient could reach the objectives of the sessions missed. Afterwards, the patient would join the experimental group again. The maximum number of sessions missed permitted was six (2 weeks).

The compliance to protocol was really good, 100% for completing training sessions with REHACOP (as can be seen in Figure 1). It must be noticed that motivation of the patients was evaluated throughout the whole process.

### Changes in neurocognition

There were no significant differences at baseline between the REHACOP and control group in total neurocognitive performance (*t=*0.83, *P*=0.410). Differences in change scores between the REHACOP and control group were found in total neurocognitive score (Table 2), indicating that REHACOP improved compared with the control group. The effect size was medium-large.

### Changes in social cognition

There were no significant differences at baseline between the REHACOP and control group in ToM (*t=*0.11, *P*=0.913), SP (*t=*1.10, *P*=0.276), EP (*t=*0.91, *P*=0.361), Managing Emotions (ME; *t=*0.21, *P*=0.834), and AS (*t=*1.50, *P*=0.138). Analysis of covariance results indicated that there were significant differences in change scores between REHACOP and control group in ToM, SP, EP, and ME. SSB (a measure of AS included in our analyses), on the other hand, was not significant. These results, along with the effect size, are shown in Table 2. The effect size was large for ToM and medium for the rest of social cognitive measures.

### Changes in clinical symptoms and functional outcome

There were no significant differences at baseline between REHACOP and the control group in positive symptoms (*t=*0.18, *P*=0.861), negative symptoms (*t=*0.55, *P*=0. 583), desorganization (*t=*0.58, *P*=0.563), excitement (*t=*0.75, *P*=0.455) and emotional distress (*t=*0.86, *P*=0.393). There were no significant differences at baseline between REHACOP and the control group in Global assessment of Functioning (GAF; *t=*0.36, *P*=0.719) and functional competence (*t=*0.41, *P*=0.680). Analysis of covariance results are displayed in Table 3. These results indicated that there were significant differences in change scores between REHACOP and control group in negative symptoms, emotional distress, GAF and functional competence. The effect size was large for functional competence and medium for GAF, negative symptoms and emotional distress.

With regard to negative symptom subfactors, we only found significant improvement in social amotivation (*F*=8.11, *P*=0.005), showing a medium effect size ($\eta_{p}^{2}=0.077$). Expressive deficits, on the other hand, did not show significant improvement after cognitive remediation (*F*=3.08, *P*=0.083). Finally, there were no significant differences in change scores between the two groups in positive symptoms, disorganization, or excitement symptoms.

## Discussion

This randomized controlled trial evaluated the efficacy of the modified version of REHACOP, which combined cognitive remediation, social cognitive intervention, and functional skills training. Patients receiving REHACOP improved significantly as compared with controls in neurocognition, social cognitive measures, negative symptoms, and functional outcome. Generally speaking, our study found positive results similar to other recently published integrative approaches,^9,12,14^ including the previous trial with the first version of REHACOP.^13^ Unfortunately, direct comparisons with these previous results with REHACOP are very difficult. The main reasons are different clinical profile of the patients, the different duration of both versions of the intervention, and differences between outcome measures (UPSA versus WHO-DAS).

Our study showed that patients receiving REHACOP improved significantly as compared with controls in three out of the four social cognitive domains evaluated. The domains that significantly improved were EP, ToM, and SP. The positive results for EP are in accordance with a recent meta-analysis.^10^ However, they found a large effect size, whereas the current study yielded a medium effect size. One of the differences between our study and other studies that found larger effect sizes in EP is the specific way in which this domain is trained. For example, our program used static pictures, whereas other programs included dynamic emotions through videos and voice recordings.^12^ Furthermore, social cognition training did not have the same length in our integrative intervention program as it did in programs that address only social cognition.^10^

Positive results in ToM are consistent with previous social cognitive remediation studies.^10^ In this case, the effect size of this study is similar to the effect size reported by Kurtz *et al.* (*d*=0.70), showing that most of the studies found significant results.^10^ In fact, a similar finding was also previously reported using the same method in patients suffering from Parkinson’s disease with ToM deficits.^20^ These generally positive results suggest that this domain is likely to be improved through cognitive interventions.

SP has not been studied in schizophrenia as thoroughly as other components of social cognition, such as EP or ToM.^21^ Our study yielded a medium effect size. These results are consistent to some extent with a handful of previous studies.^11,22^ Nevertheless, there are studies that show no evidence of improvement in SP measures^23^ after social cognitive remediation. The measure included in this study (Situational Feature Recognition Test) is different from the instruments included in the aforementioned studies, so we cannot rule out the possibility that the differences between studies are due at least in part to this circumstance. SP, as a construct, is more complex than EP. Therefore, the assessment and treatment could, in turn, present more difficulties.

AS did not show any significant improvement in our study. Kurtz *et al.*^10^ found a significant, yet small effect size in two subdomains of AS (aggression and blame) and a moderate one for hostility. The measure used in all these studies was the Ambiguous Intentions Hostility Questionnaire.^10^ The Attributional Style Questionnaire (ASQ) does not allow for such subanalyses, as it is mainly focused on the internal versus external attribution for events.

Regarding the generalization of training effects, we found improvements in negative symptoms and emotional distress. Improvement in negative symptoms is in accordance with a few other studies,^9,24^ although it has presented a hurdle in many others.^25,26^ Positive symptoms, on the other hand, did not significantly improve in our study. The lack of improvement in positive results is, nonetheless, consistent with previous similar studies.^14,27^

One of the main results of this study is the large effect size found in functional competence (UPSA). This is particularly relevant if we consider that the UPSA evaluates performance on several real world tasks simulations, rather than the patient’s or the clinician’s subjective impression of the functional outcome. Nevertheless, we also found significant improvements in global functioning, as measured by GAF. As previously mentioned, functional outcome is the last outcome target of treatments developed for patients with schizophrenia.^28^ Some possible reasons for the benefits found in functional outcome may be the group format and specific strategies, including positive feedback and homework activities. In this context, group-based interventions may be especially interesting for social cognitive and functional outcome improvements, as both rely heavily on social interactions. Therefore, interventions that are based on this—or that at least include opportunities to interact in a social context under professional supervision—may obtain greater benefits as compared with treatments that intervene at an individual level or treatments that are computer-based.

Immediate positive feedback provided by the therapist may result in positive learning experiences, boost self-confidence and self-esteem and perceived self-efficacy, which in turn may produce beneficial effects on general improvement. These variables are generally thought to be positive factors in a broader context.^11^ Nonetheless, it is not illogical to assume that part of the improvement found in negative symptomatology is due in part to these factors. The inclusion of homework activities in the therapy also supports the transfer of learned strategies to daily life activities.

Any rehabilitation treatment involves treating persons and other specific components of social cognition. Therefore, it is not surprising that including social cognition in the rehabilitation of patients with schizophrenia, allows affected persons to show improved interactions with the rehabilitation team and with their peers and colleagues in daily life. In the same way that improving memory deficits can help the patient dealing with doctor appointments or medication intake, improving social cognition can help with the therapist–patient relationship, avoiding causal–effect misunderstandings or reducing negative symptoms as observed in our study. If the patient is trained and therefore feels more confident in his/her abilities to interact, there may be a greater probability for the patient to initiate a social interaction or activity.

Our study had several limitations. First, some social cognition measures included in the study are based on paper and pencil tasks. The assessment of EP through static pictures instead of videos, for example, may not fully capture all details involved in this process. AS measured with general questions about possible attributions may also generate unreliable answers. Second, we did not include a longitudinal follow-up of the results, so the durability of the benefits obtained in this study remain unknown. Third, the negative symptoms were measured with the Positive and Negative Syndrome Scale (PANSS). Some studies published after this clinical trial was designed^29^ have suggested that this method has its limitations. To attempt to overcome them, in this study we analyzed the social amotivation and expressive deficit factors suggested by Liemburg *et al.*^30^

## Materials and methods

### Participants

The sample consisted of 111 patients diagnosed with schizophrenia, recruited from the Osakidetza Public Mental Health Services in Bizkaia (Spain). They all met the diagnostic criteria for schizophrenia according to the American Psychiatric Association’s Diagnostic and Statistical Manual of Mental Disorders, Fourth Edition, Text Revision.^31^ Exclusion criteria included evidence of alcohol or drug abuse in the past 30 days, previous history of a significant lack of consciousness, mental retardation, and relevant neurological or medical conditions. The 35.6% of patients were outpatients and 64.4% were inpatients. There were no significant differences between inpatients and outpatients in any of the change scores analyzed (*F* values ranged from 0.01 to 3.45, and *P* values ranged from 0.982 to 0.067). The study protocol was approved by the Ethics Committee at the Health Department of the Basque Mental Health System in Spain. All patients were volunteers who provided written informed consent to participate in the study. Patients were not paid to attend rehabilitation sessions.

### Design

*A priori* power analyses were conducted to determine the sample size, based on a previous study that used REHACOP. Based on previous findings,^18^ a sample size of 100 subjects, 50 in each group, was sufficient to attain an effect size of 0.57 in functional outcome between the groups, with 80% power and a 5% level of significance. The study design was a parallel-group randomized trial with equal randomization. Assignment to the program (carried out by JP) was conducted on the basis of a computer-generated randomization of the list of participants (randomization.org). Recruitment and enrollment (carried out by NO) were conducted between March 2012 and April 2014. Patients were offered the opportunity to participate in the study by their psychiatrists. Afterwards, the participants were randomly assigned to either the REHACOP group or control group (Figure 1). Post-treatment assessment was performed within the first week after completing the intervention. All raters were blind to the treatment condition and had no other role in the project that would undermine the blinding.

### Measures

Study outcome measures. The primary outcome measures were the change in mean general neurocognitive performance and social cognition (ToM, EP, AS, and SP) scores from baseline to the end of treatment.

The secondary outcome measures included change in mean clinical symptoms (Positive and Negative Syndrome Scale) and functional outcome (UCSD Performance-Based Skills Assessment and the Global Assessment of Functioning) scores from baseline to the end of treatment.

#### Neurocognition

Cognitive performance was assessed with the Hopkins Verbal Learning Test-HVLT,^32^ Digit Span, and Digit Symbol subtests from the Wechsler Adult Intelligence Scale-III^33^ and the Stroop Test.^34^ All these cognitive measures were converted into *Z*-scores based on the pooled schizophrenia group. The neurocognition composite score showed high internal consistency (Cronbach’s alpha=0.80).

Cognitive reserve was based on two measures: The Accentuation Reading Test (TAP^35^), a Spanish version of the National Adult Reading Test (NART^36^), and the Information subtest form the Weschler Adult Intelligence Scale (WAIS-III^33^). The cognitive reserve composite score showed high internal consistency (Cronbach’s alpha=0.77).

#### Social cognition

Social cognition assessment included four tests covering the four domains of social cognition: ToM, EP, SP, and AS. The Happé test^37^ was administered to evaluate ToM. Four different stories (concerning double bluffing, mistakes, persuasion, and white lies) were administered at baseline and follow-up, and they were added together to obtain a total ToM score with a possible range of 0–8. Higher scores indicate better ToM performance.

EP was evaluated using the Spanish version^38^ of Mayer-Salovery-Caruso Emotional Intelligence Test, version 2.0. Two branches were included in this study: ME and perceiving Emotions. ME asks patients to identify emotions conveyed through facial expressions and pictures. Perceiving Emotions asks patients to rate which emotional strategy would be most effective for emotional self-regulation, as well as other people’s emotions.

SP was evaluated by the Situational Feature Recognition Test.^39^ The Situational Feature Recognition Test is a paper-and-pencil measure that requires participants to identify features from a list of descriptors that describe five familiar situations (e.g., taking a test, reading in a library, and driving a car) and four unfamiliar situations (e.g., building an igloo and performing surgery). Participants are presented with a list of features for each situation, corresponding to actions, roles, rules, and goals. Each list includes six features and eight distracters.

AS was assessed using the Spanish version^40^ of the ASQ. The ASQ is a seven-point Likert scale in which participants are asked to indicate the extent to which they would attribute six hypothetical negative and positive life events to internal, stable, and global causes. Lower scores indicate the beliefs that events are caused by external, unstable, specific, or uncontrollable causes, whereas higher scores indicate the opposite. We reported one score from the ASQ: self-serving bias. This cognitive bias consists of deflecting self-blame by excessively attributing negative events to external causes and, at the same time, displaying an exaggerated tendency to attribute positive events to internal causes. SSB scores were calculated by subtracting the internality mean score for the negative events from that of the positive items. A negative score denotes a trend towards internalizing positive events and externalizing negative events. Positive SSB scores denote the opposite. Trends are more exaggerated when the scores (positive or negative) are higher.

#### Clinical symptoms

Psychopathology was assessed by means of the PANSS.^41^ PANSS was scored using a five-factor model. The five components were positive, negative, disorganization, excitement, and emotional distress (see ref. 42 for details). In addition, we created two subfactors of negative symptoms from PANSS, based on recent proposals:^30^ social amotivation and expressive deficits. More precisely, the social amotivation subfactor was based on (N2) emotional withdrawal, (N4) passive/apathetic social withdrawal, and (G16) active social avoidance. The expressive deficits subfactor was based on (N1) flat affect, (N3) poor rapport, (N6) lack of spontaneity, (G5) mannerisms and posturing, (G7) motor retardation, and (G13) avolition.

#### Functional outcome

Functional competence was evaluated with the University of California, San Diego, UCSD Performance-Based Skills Assessment—UPSA.^43^ The UPSA involves role-play tasks to assess skills in five areas: household chores, communication, finance, transportation, and planning recreational activities. The sum of these five areas (total score) was included in the analyses. Higher scores indicate better functioning.

Global functioning was based on the clinicians’ ratings of the GAF.^44^ Higher scores indicate better functioning.

### Intervention

The structure and procedure of REHACOP has been fully explained elsewhere.^18^ It includes bottom-up and top-down strategies, and its high structuration allows replication and reduces the effect of differences between therapists.

In this study, two psychologists conducted the REHACOP group, which attended 90-min sessions, 3 days per week, at the hospitals. Both psychologists used the same materials and instructions, and received the same training on REHACOP. Specifically, the REHACOP group remediation consisted of the following units: the Attention unit (4 weeks), with training on sustained, selective, alternant, and divided attention; the Memory unit (3 weeks), focusing on visual and verbal learning, recall and recognizing memory; the Language unit (3 weeks), including grammar, syntax, vocabulary, verbal fluency, verbal comprehension, and abstract language; the Executive functions unit (2 weeks), with training on cognitive planning, proverbs, and analogies; and the Social cognition unit (1 week), dedicated to the ToM, social reasoning, and moral dilemmas.

The REHACOP program includes materials to train social cognition during 1 month approximately (if administration is in group). Only 1 week of training was included as part of this clinical trial design due to time constrains. A selection of the materials was made before starting the clinical trial, as for the rest of cognitive domains. This selection included tasks related to emotion recognition (i.e., identify the six basic emotions in cards and later interpret each of these emotions on his/her own face), social reasoning (i.e., choosing the most appropriate behavior for a certain complex situation and discussing pros and cons), ToM (i.e., a history is read and described and questions about the reasons someone acted in this particular way, empathic judgments, causal–effect relationships, and alternatives are discussed), and ethics and morality (i.e., the use of white lies in social situations) applied to real-world situations. The training was based on paper and pencil tasks, as well as role playing and active group discussions. All tasks were gradually arranged in ascending difficulty.

In addition, the neuropsychologist running the groups rated the motivation of the patients to participate on a Likert-type scale, ranging from 0 (minimum motivation) to 5 (maximum motivation). Subsequently, once a patient obtained two consecutive scores below 3, the therapist provided individual feedback and encouragement and explained the reasons why cognitive remediation is needed.

The control group participated in occupational group activities led by a clinical psychologist. The activities included drawing, gardening, reading the daily news, and building things from different materials (such as paper or wood). These activities were performed in a group format and with the same frequency and timing as the implementation of REHACOP in the experimental group.

### Data analyses

IBM SPSS version 22.0 (SPSS Inc., Chicago, USA) was used for all statistical analyses. Normality of data was tested using the Shapiro–Wilk test. All variables appeared as normal distributions, with the exception of Situational Feature Recognition Test and positive symptoms, which were log-transformed for further analyses. Categorical data were analyzed using the *χ*^2^-test. Sociodemographic variables, clinical variables, cognition, and functional outcome at baseline were compared using two-tailed *t*-tests.

Changes in scores (post treatment–baseline) were compared between the REHACOP and control group for each of the cognitive, clinical, and functional disability controlling for baseline scores (analysis of covariance). Partial eta squared $\left(\eta_{p}^{2}\right)$ was obtained as an indicator of the effect size and it was interpreted as small, medium, and large based on values of 0.01, 0.06, and 0.14, respectively.^45^ Significance level was set at 0.05. All tests were two-tailed.

## Figures and Tables

**Table 1:** Participant characteristics at baseline

	*REHACOP group (*n*=52)*		*Control group (*n*=49)*		P *value*
	*Mean (95% CI)*	*s.d.*	*Mean (95% CI)*	*s.d.*	
Age (years)	39.87 (37.23 to 42.50)	9.5	38.13 (36.54 to 42.36)	10.1	0.831
Years of education (years)	10.55 (9.26 to 11.09)	3.29	10.25 (8.99 to 10.73)	3.0	0.618
Age at the onset (years)	24.13 (22.16 to 26.11)	7.1	22.29 (20.52 to 24.07)	6.1	0.169
Number of hospitalizations	8.10 (6.00 to 10.20)	7.5	7.08 (4.58 to 9.59)	8.7	0.532
					
*Gender:* n *(%)*					
Males	36 (75.5%)	37 (69.2%)			0.481
Females	16 (24.5%)	12 (30.8%)			
					
*DSM-IV-TR:* n *(%)*					
Paranoid	35 (67.3%)	33 (67.4%)			0.411
Disorganized	4 (7.7%)	8 (16.3%)			
Residual	5 (9.6%)	2 (4.1%)			
Non-specified	8 (15.4%)	6 (12.2%)			
Cognitive reserve index	0.09 (−0.16 to 0.14)	0.8	−0.11 (−0.34 to 0.12)	0.8	0.196
Chlorpromazine equivalent doses (mg per day)	497.65 (435.9 to 559.4)	227.7	479.66 (402.7 to 556.7)	275.7	0.755

Abbreviations: CI, confidence interval; Cognitive reserve index, Cognitive reserve composite score; DSM-IV-TR, The Diagnostic and Statistical Manual of Mental Disorders 4th edition, Text Revised.

**Table 2:** Cognitive and social cognitive performance in the REHACOP group and control group at baseline and post treatment

	*REHACOP group*		*Control group*		*ANCOVA for change scores*		*Effect size*
	*Mean (95% CI)*	*s.d.*	*Mean (95% CI)*	*s.d.*	F	P *value*	$\eta_{p}^{2}$
*nps*							
Pre	−0.06 (−0.27 to 0.15)	0.8	0.06 (−0.15 to 0.28)	0.8			
Post	0.08 (−0.13 to 0.29)	0.7	−0.09 (−0.29 to 0.13)	0.8			
Change scores	0.14 (0.04 to 0.24)	0.4	−0.15 (−0.25 to −0.05)	0.4	15.73	<0.001	0.138
							
*ToM*							
Pre	3.40 (2.82 to 3.99)	2.1	3.45 (2.86 to 4.04)	2.1			
Post	4.88 (4.39 to 5.38)	2.8	3.59 (2.93 to 4.25)	2.3			
Change scores	1.47 (1.03 to 1.92)	2.1	0.15 (−0.30 to 0.61)	1.3	17.03	<0.001	0.148
							
*SP*							
Pre	68.29 (64.25 to 72.33)	14.5	63.90 (56.93 to 70.86)	24.2			
Post	71.20 (67.50 to 74.89)	13.1	62.72 (55.88 to 69.55)	23.2			
Change scores	2.70 (−0.79 to 6.20)	8.7	−4.71 (−8.35 to −1.07)	15.8	8.50	0.004	0.082
							
*EP*							
Pre	96.50 (92.27 to 100.73)	14.8	96.74 (92.46 to 101.01)	15.3			
Post	102.42 (97.85 to 106.99)	15.8	95.82 (91.20 to 100.44)	16.8			
Change scores	5.89 (2.39 to 9.38)	12.3	−0.88 (−4.41 to 2.65)	13.9	7.31	0.008	0.071
							
*ME*							
Pre	88.72 (85.80 to 91.64)	10.7	88.96 (86.01 to 91.91)	10.1			
Post	90.92 (88.04 to 93.80)	11.1	86.98 (83.26 to 90.70)	9.3			
Change scores	2.14 (−0.38 to 4.66)	9.7	−2.57 (−5.12 to −0.02)	11.1	6.80	0.011	0.066
							
*AS*							
Pre	0.92 (0.54 to 1.28)	1.3	0.55 (0.22 to 0.87)	1.1			
Post	0.91 (0.54 to 1.28)	1.3	0.78 (0.46 to 1.10)	1.1			
Change scores	0.14 (−0.22 to 0.49)	1.9	0.08 (−0.28 to 0.43)	1.2	0.06	0.811	0.001

Abbreviations: $\eta_{p}^{2}$, partial eta squared; AS, attributional style, ASQ; ANCOVA, analysis of covariance; CI, confidence interval; Change scores, covariance adjusted means of change scores; EP, Perceiving Emotions branch from MSCEIT; ME, Managing Emotions branch from MSCEIT; MSCEIT, Mayer-Salovery-Caruso Emotional Intelligence Test, version 2.0; nps, Neurocognitive composite score; SP, social perception, SFRT; SFRT, Situational Feature Recognition Test; ToM, theory of mind, Happé.

**Table 3:** Clinical characteristics and functional disability in the REHACOP and control group at baseline and post treatment

	*REHACOP group*		*Control group*		*ANCOVA for change scores*		*Effect size*
	*Mean (95% CI)*	*s.d.*	*Mean (95% CI)*	*s.d.*	F	P *value*	$\eta_{p}^{2}$
*Positive*							
Pre	13.83 (11.91 to 15.73)	6.5	14.07 (12.15 to 15.96)	6.6			
Post	10.28 (8.83 to 11.74)	4.9	10.52 (9.07 to 1.98)	5.0			
Change scores	−3.58 (−3.58 to −2.72)	4.1	−3.42 (−4.32 to −2.52)	4.3	0.07	0.783	0.001
							
*Negative*							
Pre	21.61 (18.87 to 24.35)	9.4	21.65 (18.91 to 24.40)	9.3			
Post	16.17 (13.94 to 18.40)	6.9	18.80 (16.57 to 21.04)	8.3			
Change scores	−5.29 (−6.45 to −4.13)	6.4	−2.82 (−4.01 to −1.62)	4.4	8.60	0.004	0.082
							
*Disorganization*							
Pre	12.83 (11.33 to 14.32)	5.3	12.96 (11.46 to 14.46)	4.9			
Post	9.72 (8.52 to 10.91)	3.9	9.72 (10.02 to 12.41)	3.9			
Change scores	−2.98 (−3.76 to −2.21)	4.0	−1.69 (−2.49 to −0.89)	3.4	3.70	0.057	0.052
							
*Excitement*							
Pre	9.33 (7.99 to 10.65)	4.8	8.41 (7.09 to 9.74)	4.2			
Post	7.30 (6.36 to 8.25)	3.4	7.00 (6.06 to 7.94)	3.0			
Change scores	−1.73 (−2.36 to −1.11)	3.5	−1.55 (−2.19 to −0.90)	2.8	0.17	0.683	0.002
							
*Emotional distress*							
Pre	10.48 (9.45 to 11.51)	6.5	10.48 (8.75 to 10.81)	6.6			
Post	7.67 (6.77 to 8.58)	2.9	9.04 (8.14 to 9.95)	3.3			
Change scores	−2.68 (−3.33 to −2.02)	3.5	−0.81 (−1.49 to −0.14)	2.4	12.73	0.001	0.136
							
*GAF*							
Pre	42.24 (38.23 to 46.24)	12.6	43.09 (39.08 to 47.09)	14.6			
Post	56.02 (51.51 to 60.53)	17.0	50.46 (45.95 to 54.96)	5.4			
Change scores	13.27 (10.25 to 16.28)	15.6	6.95 (3.84 to 10.05)	8.9	8.36	0.005	0.081
							
*Functional competence*							
Pre	68.84 (64.83 to 72.86)	14.2	67.65 (63.51 to 71.78)	14.9			
Post	76.05 (72.10 to 80.00)	12.8	68.71 (64.64 to 72.78)	15.8			
Change scores	8.24 (5.63 to 10.85)	9.1	0.94 (−1.64 to 3.52)	9.0	15.61	<0.001	0.154

Abbreviations: $\eta_{p}^{2}$, partial eta squared; ANCOVA, analysis of covariance; CI, confidence interval; Change scores, covariance adjusted means of change scores; Disorganization, Disorganized symptoms from PANSS; Emotional dis, Emotional distress from PANSS; Excitement, Excitement symptoms from PANSS; GAF, Global Assessment of Functioning; Negative, Negative symptoms from PANSS; Positive, Positive symptoms from PANSS; UPSA, UCSD Performance-Based Skills Assessment.
